# Impact of Disease‐Specific Treatment and Non‐Selective Beta Blockers on Risk of PVT in Cirrhotic Patients With HCV or PBC

**DOI:** 10.1111/liv.70628

**Published:** 2026-04-03

**Authors:** Humberto C. Gonzalez, Stuart C. Gordon, Sheri Trudeau, Trueman Wu, Lora Rupp, Christina Melkonian, Mark A. Schmidt, Yihe G. Daida, Amandeep K. Sahota, Christopher L. Bowlus, Mei Lu

**Affiliations:** ^1^ Division of Gastroenterology and Hepatology Henry Ford Health Detroit Michigan USA; ^2^ School of Medicine Wayne State University Detroit Michigan USA; ^3^ College of Human Medicine Michigan State University East Lansing Michigan USA; ^4^ Center for Health Policy and Health Services Research Henry Ford Health Detroit Michigan USA; ^5^ Department of Public Health Sciences Henry Ford Health Detroit Michigan USA; ^6^ Center for Health Research Kaiser Permanente Northwest Portland Oregon USA; ^7^ Center for Integrated Health Care Research Kaiser Permanente Hawaii Honolulu Hawaii USA; ^8^ Department of Research and Evaluation Kaiser Permanente Southern California Los Angeles California USA; ^9^ Division of Gastroenterology and Hepatology University of California Davis California USA

**Keywords:** AASLD, decompensated cirrhosis, statins, sustained virological response (SVR), ursodeoxycholic acid (UDCA)

## Abstract

**Background:**

Portal vein thrombosis (PVT) is a common sequela of cirrhosis. Despite regression of fibrosis/cirrhosis observed among some patients after disease‐specific treatment, few studies have considered the role of treatment and response on risk of PVT among patients with cirrhosis across different liver disease states. We considered aetiological treatment and response as well as whether non‐selective beta‐blockers (NSBBs), statins, and anticoagulants were associated with risk of PVT.

**Methods:**

We used data for patients with cirrhosis (compensated or decompensated) from two large, US‐based multisite cohort studies. Patients with hepatitis C (HCV) were drawn from the Chronic Hepatitis Cohort Study. Patients with primary biliary cholangitis (PBC) were drawn from the Fibrotic Liver Disease Consortium. Risk for PVT was assessed using a discrete survival model that includes both fixed covariates and time‐dependent variables.

**Results:**

Among 6659 HCV patients, 274 developed PVT across ~13 years of follow‐up. Significant risk factors included time‐varying decompensated cirrhosis (adjusted hazard ratio [aHR] 27.41, 95% confidence interval [CI] 15.89–47.30), male sex (aHR 1.48, 95% CI 1.12–1.94), and use of NSBBs (aHR 2.07, 95% CI 1.61–2.67). Among 786 PBC patients, 67 developed PVT across ~8 years of follow‐up. Use of NSBBs was the only significant PVT risk factor in these patients (aHR 2.56, 95% CI 1.52–4.31). Neither sustained virological response [SVR] after antiviral treatment (HCV patients) nor response to ursodeoxycholic acid [UDCA] treatment (PBC patients) was associated with risk of PVT.

**Conclusions:**

In two large well‐characterized samples of cirrhotic patients with HCV or PBC, disease‐specific treatments were not associated with PVT risk. NSBB treatment was independently associated with > 2 times the risk of PVT in both samples, regardless of compensated or decompensated status.

## Keypoints

- We did not observe an association between risk of PVT and SVR after antiviral treatment for hepatitis C (HCV).
- Among HCV patients, decompensated cirrhosis and male sex were associated with higher rates of PVT.
- We did not observe an association between risk of PVT and treatment with ursodeoxycholic acid for primary biliary cholangitis (PBC).
- Receipt of non‐selective beta blockers was associated with higher rates of PVT in both HCV and PBC patients.

**Abbreviations**
aHR: adjusted hazard ratio
BMI: body mass index
CCI: Charlson Comorbidity Index
CHeCS: Chronic Hepatitis Cohort Study
CI: confidence interval
FIB4: Fibrosis‐4 index
FOLD: Fibrotic Liver Disease
GEE: generalized estimating equations
HCV: hepatitis c virus
ICD: International Classification of Diseases
IFN: interferon
IPTW: inverse probability of treatment weighting
NSBB: non‐selective beta blockers
PBC: primary biliary cholangitis
PVT: portal vein thrombosis
SVR: sustained virological response
T2D: type 2 diabetes
TF: treatment failure
UDCA: ursodeoxycholic acid
ULN: upper limit of normal

## 1 Introduction

Portal vein thrombosis (PVT)—a blood clot causing narrowing or blockage of the portal vein—is a common sequela of liver cirrhosis. Reported occurrence of PVT varies by method of evaluation and cause of cirrhosis [1], but a meta‐analysis found prevalence of roughly 14% and lifetime incidence of 10% among patients with cirrhosis [2]. This analysis also identified a number of risk factors, including advanced and decompensated cirrhosis as well as use of non‐selective beta blockers (NSBBs).

Despite the central role of cirrhosis as a precursor to PVT, few studies consider the impact of treatment for underlying liver disease with regard to PVT risk. For example, sustained virological response (SVR) to antiviral treatment for hepatitis C (HCV) is associated with reductions in measures of hepatic vein pressure and liver stiffness [3, 4], which may be expected to reduce risk of PVT. However, a recent review reported that few studies have considered the role of disease‐specific treatment in addressing risk of PVT [5], although one study found no impact of SVR on short‐term risk of PVT among HCV patients [6]. Likewise, despite evidence of increased coagulation even in the early stages of primary biliary cholangitis (PBC) and evidence that ursodeoxycholic acid (UDCA) treatment prevents the progression of portal hypertension [7], there are no studies specifically considering the impact of UDCA treatment on risk of PVT.

To address this knowledge gap, we leveraged data from two large US‐based cohort studies of HCV and PBC—the Chronic Hepatitis Cohort Study (CHeCS) and the Fibrotic Liver Disease Consortium (FoLD)—to evaluate the impact of aetiological treatment on risk of PVT among our racially and geographically diverse samples of patients with cirrhosis. We also considered the role of patient demographic and clinical characteristics, as well as treatment with NSBBs, anti‐coagulants, and statins.

## 2 Methods

### 2.1 Cohort Selection

We used data for patients with cirrhosis (compensated or decompensated) from two large, multisite cohort studies. HCV patients were drawn from the US‐based Chronic Hepatitis Cohort Study (CHeCS) [8]. CHeCS is a retrospective/prospective, observational study of viral hepatitis patients from four large health systems Geisinger Health System (Danville PA); Henry Ford Health System (Detroit MI); Kaiser Permanente Hawaii (Honolulu HI); and Kaiser Permanente Northwest (Portland OR). PBC patients were drawn from the Fibrotic Liver Disease (FOLD) Consortium, which comprises 11 geographically‐diverse health systems [9, 10]. This analysis used updated data through 2021 from five FOLD sites: Geisinger Health System (Danville PA), Henry Ford Health (Detroit MI), Kaiser Permanente Hawaii (Honolulu HI), Kaiser Permanente‐Northwest (Portland OR), and Kaiser Permanente‐Southern California (Los Angeles CA).

Both CHeCS and FOLD follow the guidelines of the US Department of Health and Human Services for the protection of human subjects. Study protocols were approved by the Institutional Review Board at Henry Ford Health (IRB No. 5953) and at each study site. Requirements for written informed consent were waived due to the observational and de‐identified nature of the data. All authors had access to the study results and reviewed and approved the final manuscript.

Detailed methods for patient identification have been previously described for both CHeCS and FoLD [8, 9, 10]. Diagnoses of HCV or PBC were confirmed with chart abstraction. Patients from both cohorts were considered for inclusion in this analysis if the date of cirrhosis diagnosis was after the date of the disease diagnosis. For HCV patients, we identified cirrhosis using a previously published hierarchical hybrid algorithm [11] to identify cirrhotic patients based on multiple sources: (1) decompensated cirrhosis identified using a validated hybrid model and confirmed via chart review by trained medical abstracters; (2) “F4” liver biopsy or transient elastography results > 12.5 kPa [12]; (3) FIB4 > 5.88 [13]; and (4) presence of International Classification of Diseases [ICD] 9/10 diagnosis codes for cirrhosis in the electronic health record. For PBC patients, we identified cirrhosis using: (1) decompensated cirrhosis identified by our previously published Classification and Regression Tree [CART] model [14]; (2) liver biopsy fibrosis classification of F4; (3) a transient elastography liver stiffness score > 17; (4) an Lscore ≥ 0.1 [14]. For both groups, patients who met criteria 1 were classified as having decompensated cirrhosis; patients who did not meet criteria 1 but met at least one of criteria 2–4 were classified as having compensated cirrhosis. Patients were excluded if they were co‐infected with hepatitis B, had received a liver transplant prior to their index date, or had been diagnosed with PVT prior to their index date. For both cohorts, index dates were defined as the date of initiation of first disease‐related treatment (e.g., antiviral treatment for HCV or UDCA for PBC); for never‐treated patients, index dates were defined as the first date of cirrhosis diagnosis. PVT events were identified using ICD‐CM codes 181 (ICD9) and 452 (ICD10).

Data regarding HCV treatments (interferon [IFN] or direct‐acting antivirals [DAA]) was abstracted from electronic medical records, including drug names, and start/stop dates for each course of medication. For PBC patients, ursodeoxycholic acid (UDCA) treatment data was collected via chart abstraction. After initiation of UDCA therapy, patients were assumed to be on continuous treatment. Time‐dependent variables for both cohorts included: cirrhosis status (compensated vs. decompensated); treatment with non‐selective beta‐blockers [NSBB], anti‐coagulants/platelets, or statins (detailed in Table S1); type 2 diabetes ([T2D] yes/no); and Charlson Comorbidity Index (CCI, excluding liver‐related comorbidities) as well as fixed covariates (age at index; sex; race; and histories of thrombotic events, malignant neoplasms, pregnancy/hormone therapy, and major abdominal surgery). For the HCV cohort, antiviral treatment status/response (sustained virological response [SVR], treatment failure [TF], untreated, ongoing treatment) and BMI were also included as time‐varying covariates. For the PBC cohort, UDCA treatment (yes/no) was included as a static variable and treatment response (defined as alkaline phosphatase [ALP] < 1.67 times the upper limit of normal [xULN]) was included as a time‐varying covariate.

### 2.2 Statistical Analysis on Modelling Discrete Time‐To‐Event Data

PVT was the outcome of interest. Patients were followed from index date until an event of interest, death, or the end of follow up. Death was considered a competing risk in the analyses; it was ascertained using electronic health record data and a search against national/state death indices. Receipt of liver transplant was used as a censoring event in the analysis. For each patient, observation time (noted as ‘X’) was calculated as years from the index date to the first event of: (1) PVT diagnosis date; (2) liver transplant or death; and (3) date of last follow‐up (up to 13 years for HCV and 8 years for PBC cohort post‐index).

Separate analyses were performed for each cohort due to differences in sample characteristics and data structure. Risk for PVT was assessed using a previously described discrete survival model [15, 16] that includes both fixed covariates and time‐dependent variables, and a ‘pseudo‐observations’ approach [17] to compress data into discrete time intervals using 1 year as the landmark interval (INT), where each ‘INT’ has a value of 0 (at baseline) through the years post‐index or the date of last follow‐up. To address treatment selection bias, we used a time‐varying propensity score approach to estimate inverse probability of treatment weighting (IPTW) based on logistic regression; for HCV patients, the outcome variable was treatment presence or absence at a given interval. Covariates measured prior to that interval included demographic variables and clinical risk factors. No weighting was applied to treatment‐ineligible patients (e.g., HCV patients categorized as having ongoing treatment from a previous interval, or who had previously achieved SVR). In the propensity weighting for PBC patients, UDCA was assumed to be continuous after initiation (as a fixed covariate); as a result, a fixed IPTW was calculated at index date and then adjusted in the multivariable analysis. Standardized mean differences were used to examine the balance of variable distribution between treatment groups before and after IPTW at each interval.

The model included both fixed and time‐varying covariates to estimate the effects of disease‐specific treatment/response on risk of PVT after adjusting time‐varying propensity scores for treatment selection bias. We used generalized estimating equations (GEE) with the multinominal link function for discrete time‐to‐event data. The analysis began by testing individual variable effects on risk of PVT, including variable‐by‐treatment interactions, followed by the multivariable modeling. Variables were retained in the final multivariable model if there were significant individual variable effects or variable‐by‐treatment interactions (*p*‐values < 0.05). The cause‐specific discrete hazard ratios (HR) and 95% confidence interval (95% CI) were estimated for each covariate retained in the final model.

## 3 Results

### 3.1 HCV Cohort

Table 1a displays patient demographic and clinical characteristics at index date; a total of 6659 HCV patients with cirrhosis were included and 274 patients developed PVT across roughly 13 years of follow‐up. Roughly two‐thirds of patients in the sample were male and 82% of patients were at least 50 years old; one‐quarter of the sample was Black, 62% were white, and 6% were AAPI. Table 1b displays a snapshot of sample characteristics at different lengths of follow‐up; at baseline, 27% of patients had received antiviral treatment; this proportion was 68% among patients with at least 5 years of follow‐up, and 59% among patients with 10 years of follow‐up, reflecting the lower rates of antiviral treatment receipt prior to the wide availability of DAA therapies. Across the study period, 38%–42% of the sample had decompensated cirrhosis; the proportion of patients with CCI scores ≥ 3 increased from 24% at baseline to 36% among patients with 10 years of follow‐up. The proportions of patients receiving NSBBs, anticoagulants, and statins also increased with follow‐up time (from 14%–22%, 30%–44%, and 32%–50%, respectively).

**TABLE 1:** Static variables measured at baseline by PVT outcomes (A) and a snapshot of the sample displaying time‐varying covariates measured at baseline, and at years 5 and 10 of follow‐up (B) among cirrhotic patients with hepatitis C.

A. Static variables measured at baseline				
Variable	Response	All	No PVT	PVT
		(*n* = 6659)	(*n* = 6385)	(*n* = 274)
Age	< 40	244 (4%)	236 (4%)	8 (3%)
	40 – < 50	964 (14%)	918 (14%)	46 (17%)
	50 – < 60	2775 (42%)	2650 (42%)	125 (46%)
	≥ 60	2676 (40%)	2581 (40%)	95 (35%)
Sex	Female	2313 (35%)	2239 (35%)	74 (27%)
	Male	4346 (65%)	4146 (65%)	200 (73%)
Race	White	4142 (62%)	3952 (62%)	190 (69%)
	Black	1643 (25%)	1590 (25%)	53 (19%)
	AAPI/other	410 (6%)	393 (6%)	17 (6%)
	Unknown	464 (7%)	450 (7%)	14 (5%)
History of	Malignancy	743 (11%)	714 (11%)	29 (11%)
	Thrombophilia	179 (3%)	173 (3%)	6 (2%)
	Abdominal surgery	140 (2%)	131 (2%)	9 (3%)
	Hormone treatment	158 (2%)	155 (2%)	3 (1%)
	Ectomies	58 (1%)	57 (1%)	1 (0%)

B. Time‐varying covariates at timepoints of interest				
Variable	Response	Baseline	Year 5	Year 10
Cirrhosis	Compensated	3992 (60%)	1717 (62%)	440 (58%)
	Decompensated	2667 (40%)	1046 (38%)	316 (42%)
Treatment status	SVR	551 (8%)	1348 (49%)	261 (35%)
	TF	1260 (19%)	524 (19%)	183 (24%)
	Untreated	4828 (73%)	873 (32%)	310 (41%)
	Ongoing	20 (0%)	18 (1%)	2 (0%)
Charlson comorbidity index	0	3114 (47%)	920 (33%)	264 (35%)
	1	1141 (17%)	544 (20%)	137 (18%)
	2	774 (12%)	336 (12%)	85 (11%)
	3	1630 (24%)	963 (35%)	270 (36%)
BMI	< 25	801 (21%)	45 (16%)	501 (20%)
	25 – < 30	1099 (29%)	61 (22%)	507 (20%)
	≥ 30	1365 (36%)	75 (27%)	563 (22%)
	Missing	552 (14%)	93 (34%)	997 (39%)
Diabetes	Yes	2310 (35%)	1271 (46%)	384 (51%)
NSBB	Yes	903 (14%)	592 (21%)	166 (22%)
Statins	Yes	1014 (15%)	686 (25%)	207 (27%)
Anticoagulants	Yes	2012 (30%)	1191 (43%)	328 (43%)

Abbrevations: AAPI, asian american/pacific islander; BMI, body mass index; NSBB, non‐selective beta‐blockers; PVT, portal vein thrombosis; SVR, sustained virological response; TF, treatment failure.

**FIGURE 1:**
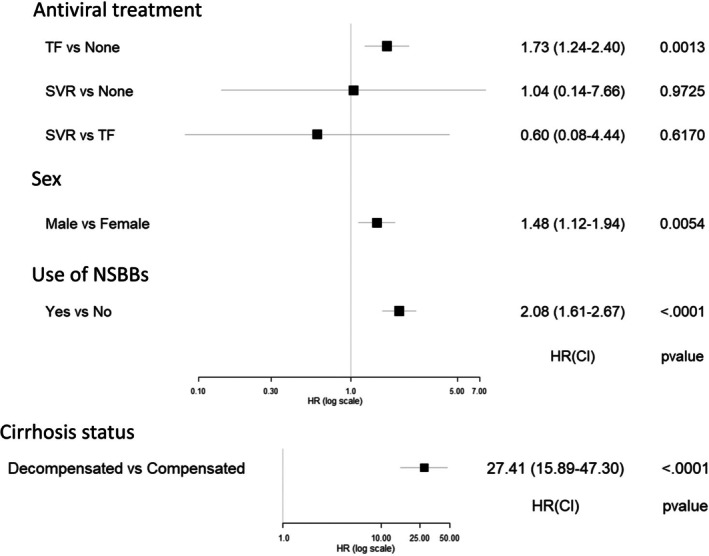
Adjusted hazard ratios and 95% confidence intervals for multivariable analysis of risk of PVT among cirrhotic patients with hepatitis C virus (note scale difference for cirrhosis).

As shown in Figure 1, time‐varying decompensated cirrhosis status was by far the strongest risk factor for PVT, associated with almost 27‐fold risk compared to compensated cirrhosis (aHR 27.41, 95% confidence interval (CI) 15.89–47.30). Male patients were at roughly 50% higher risk of PVT compared to female patients (aHR 1.48, 95% CI 1.12–1.94). With regard to disease‐specific treatment, there was no significant impact of SVR after antiviral therapy on risk of PVT compared to patients who were untreated or those who experienced treatment failure (TF); however, risk of PVT was higher in patients with TF compared to those who were untreated (aHR 1.72, 95% CI 1.24–2.40). Use of NSBBs was associated with roughly double the risk of PVT (aHR 2.07, 95% CI 1.61–2.67).

### 3.2 PBC Cohort

Table 2a displays patient demographic and clinical characteristics at index date; a total of 786 patients with cirrhosis were included and 67 patients developed PVT across roughly 8 years of follow‐up. The PBC sample was more homogeneous than the HCV sample; 90% of patients were aged 51 or older, 82% were women, and the majority of patients (72%) were white. A larger proportion of patients had decompensated cirrhosis at baseline (94%); as shown in Table 2b, that proportion decreased slightly to 88% among patients with 8 years of follow‐up. Across the study period, the proportion of the sample with CCI scores ≥ 3 increased from 34% at baseline to 47% among patients with at least 8 years of follow‐up. The proportions of patients on NSBB, anticoagulants, and statins also increased with follow‐up (from 38%–49%, 33%–47%, and 44%–66%, respectively).

**TABLE 2:** Static variables measured at baseline by PVT outcomes (A) and a snapshot of the sample displaying time‐varying covariates measured at baseline, and at years 4 and 8 of follow‐up (B) among cirrhotic patients with primary biliary cholangitis.

A. Static variables measured at baseline				
Variable	Response	All	No PVT	PVT
		(*n* = 786)	(*n* = 719)	(*n* = 67)
Age	< 40	21 (3%)	18 (3%)	3 (4%)
	40 – < 50	57 (7%)	50 (7%)	7 (10%)
	50 – < 60	200 (25%)	176 (24%)	24 (36%)
	≥ 60	508 (65%)	475 (66%)	33 (49%)
Gender	Women	645 (82%)	589 (82%)	56 (84%)
	Men	141 (18%)	130 (18%)	11 (16%)
Race	White	567 (72%)	523 (73%)	44 (66%)
	Black	43 (5%)	40 (6%)	3 (4%)
	AAPI/Others	58 (7%)	53 (7%)	5 (7%)
	Unknown	118 (15%)	103 (14%)	15 (22%)
Received UDCA	Yes	658 (84%)	599 (83%)	59 (88%)
History of	Malignancy	72 (9%)	68 (9%)	4 (6%)
	Thrombophilia	20 (3%)	19 (3%)	1 (1%)
	Abdominal surgery	39 (5%)	36 (5%)	3 (4%)
	Hormone treatment	21 (3%)	21 (3%)	0 (0%)
	Ectomies	23 (3%)	22 (3%)	1 (1%)

B. Time‐varying covariates at timepoints of interest				
Variable	Response	Baseline	Year 4	Year 8
Cirrhosis	Compensated	44 (6%)	28 (11%)	13 (12%)
	Decompensated	742 (94%)	232 (89%)	96 (88%)
ALP	≤ 1.67 × ULN	465 (59%)	124 (48%)	46 (42%)
	> 1.67 × ULN	321 (41%)	136 (52%)	63 (58%)
Charlson comorbidity index	0	236 (30%)	54 (21%)	29 (27%)
	1	147 (19%)	39 (15%)	11 (10%)
	2	134 (17%)	55 (21%)	17 (16%)
	3	269 (34%)	112 (43%)	52 (48%)
Diabetes	Yes	271 (34%)	102 (39%)	51 (47%)
NSBBs	Yes	302 (38%)	132 (51%)	53 (49%)
Statins	Yes	242 (31%)	89 (34%)	39 (36%)
Anticoagulants	Yes	255 (32%)	103 (40%)	51 (47%)

Abbrevations: AAPI, asian american/pacific islander; ALP, alkaline phosphatase; NSBB, non‐selective beta‐blockers, PVT, portal vein thrombosis; UDCA, ursodeoxycholic acid.

In contrast to the HCV cohort, the only significant risk factor for PVT in PBC patients was use of NSBBs. Use of NSBBs was associated with more than double the risk of PVT (aHR 2.56, 95% CI 1.52–4.31). Treatment with UDCA, anticoagulants, and statins was not associated with the risk of PVT in this PBC cohort.

### 3.3 Sensitivity Analyses

To address possible confounding by indication due to the relationship between oesophageal varices and receipt of NSBBs, we performed sensitivity analyses that included presence/absence of varices as a variable. We also performed an analysis that included platelet levels as a variable, reflecting the relationship between increased portal hypertension and thrombocytopenia. For both the HCV and PBC samples, the association between use of NSBBs and increased risk of PVT remained significant when varices and platelets were included in these analyses. To address the lack of granular data regarding cirrhosis severity beyond the compensated/decompensated categories used, we next performed an additional sensitivity analysis that included albumin, bilirubin, and platelets as variables related to cirrhosis progression; results were similar to those of the main analysis. Finally, to address the possibility that hepatocellular carcinoma (HCC) could be misclassified as a thrombus, we performed an analysis in the HCV sample that excluded all patients with HCC. Results were unchanged from the main analysis. There were too few diagnoses of HCC in the PBC sample to perform such an analysis.

### 3.4 Relative Incidence of PVT Between HCV and PBC Patients

**FIGURE 2:**
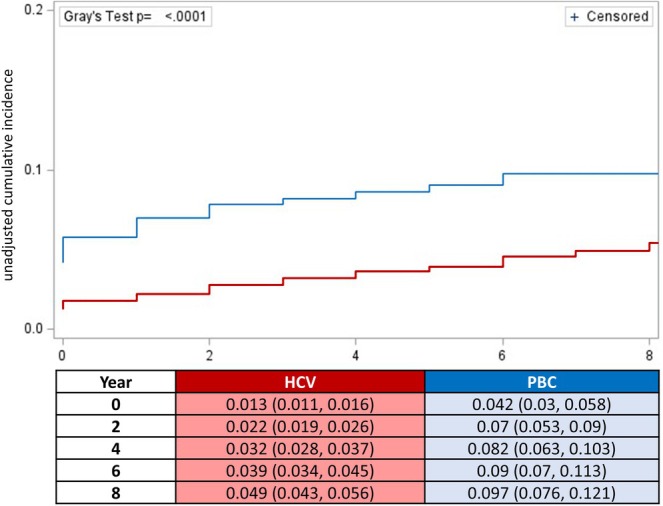
Crude (unadjusted) cumulative incidence of PVT among cirrhotic patients with hepatitis C or primary biliary cholangitis across 8 years of observation.

Although differences in the design and data structure of the two cohorts preclude formal comparisons of PVT risk factors across disease aetiology, we observed some differences in the presentation of PVT between the two groups. As shown in Figure 2, unadjusted cumulative incidence of PVT across eight years of observation was significantly higher among PBC patients compared to those with HCV (7.6% versus 4.9%, *p* < 0.0001). We note, however, the proportion of patients with decompensated cirrhosis was much higher in our PBC cohort compared to the HCV cohort.

## 4 Discussion

Using data from two large retrospective samples of patients with cirrhosis subsequent to HCV or PBC, we sought to identify risk factors for PVT after adjusting for extrahepatic conditions (abdominal surgery, thrombophilia) that may precipitate clotting. We found that treatment with NSBBs was an independent risk factor in both cohorts, associated with twice the risk of PVT in each group (aHR 2.07 [HCV] and 2.55 [PBC]) regardless of decompensated or compensated status. This finding is consistent with a recent meta‐analysis showing that use of NSBBs is associated with increased risk of PVT after adjusting for manifestations of decompensation (such as oesophageal varices) [2].

Notably, however, treatment for liver disease aetiology did not appear to impact PVT risk in either cohort. Among patients with HCV, achievement of SVR was not associated with any difference in risk of PVT, although treatment failure was associated with higher risk than no treatment; this is possibly due to the association between more severe liver disease and risk of treatment failure in the era of interferon‐based treatments. Likewise, among patients with PBC, neither UDCA treatment nor response to treatment (as defined by time‐varying ALP relative to 1.67 times the upper limit of normal) was associated with any significant change in risk of PVT.

Among patients with HCV, we found that decompensated cirrhosis was by far the strongest risk factor for PVT, increasing risk by 27 times compared to compensated cirrhosis. The relationship is well‐established in the literature. Male sex was also associated with roughly 50% higher risk of PVT. This is consistent with a large body of research showing that men are at overall higher risk of venous thrombosis from any cause compared to women [18]. In contrast, decompensation was not a risk factor among patients with PBC, although this observation is likely due to the small proportion of patients with compensated cirrhosis in this sample (44 at baseline and 13 at 8 years of follow‐up), resulting in a lack of power to detect statistical differences.

Treatment of oesophageal varices is a common indication for use of NSBBs among patients with cirrhosis. Varices were not included in the primary model due to overlap between our identification of decompensated cirrhosis and evidence of bleeding varices. However, to address possible confounding by indication, we performed sensitivity analyses that included presence/absence of varices as a variable. In both the HCV and PBC cohorts, receipt of NSBBs remained a significant risk factor for PVT even when the presence of varices was included in the model. In addition, because we do not have direct measurements of portal vein pressure or velocity, we performed sensitivity analyses that included platelet counts as a proxy for portal hypertension. As in the previous sensitivity analysis, the use of NSBBs remained significantly associated with the risk of PVT after controlling for platelet counts in both samples. This finding is consistent with a recent meta‐analysis that showed NSBBs were associated with roughly 2–3 times higher odds of PVT [2]. Additional controlled prospective studies are needed to determine whether a causal relationship exists between the use of NSBBs and incident PVT.

Our study was not designed to evaluate potential mechanisms underlying our findings. However, at least one researcher has speculated that use of NSBBs may result in slower portal inflow velocity [19], which has been associated with increased risk of PVT development [2]. To address this, clinicians may consider use of pre‐treatment imaging to determine whether a patient is at heightened risk of this outcome. Portal vein diameter greater than 12.5mm [20] and portal vein velocity < 15 cm/s [21] are independently associated with increased risk of PVT. Establishment of a patient's risk profile could guide decisions regarding the risk/benefit ratio of NSBB therapy, the frequency of patient monitoring, use of band ligation, or other strategies. Likewise, future prospective studies may consider including such measures to better interrogate the relationship between use of NSBBs and risk of PVT. In addition, although prophylactic anticoagulant therapy with enoxaparin has been shown to reduce risk of incident PVT in a clinical trial, [22] most studies related to use of anticoagulants and PVT have considered treatment of existing PVT and/or secondary prophylaxis [23, 24]. We did not observe an association with anticoagulant/antiplatelet treatment and risk of primary PVT in our samples; it is possible that differences in medication type, dose, or duration limited our ability to observe any such association.

There are unavoidable limitations to our study. Due to differences in data structure for each of the studies, we are unable to combine the cohorts and adjust for differences in sample characteristics; as a result, we cannot perform an adjusted analysis comparing incidence of and risk factors for PVT across liver disease aetiology. We have attempted to address this by considering cumulative incidence, but those results must be interpreted with caution. Moreover, the observed incidence of PVT in our cohorts is lower than that reported in the literature. We note, however, that many studies of PVT epidemiology do not consider aetiology of the underlying liver disease. Our reliance upon diagnosis codes for the identification of PVT may have resulted in missed events or misclassification; however, our findings are roughly consistent with those of two studies of PVT epidemiology among HCV patients who are not listed for liver transplant [6, 24]; we are not aware of any studies that reported incidence of PVT among patients with PBC. We also do not have measures of liver disease severity like MELD score or Child‐Pugh stage, nor direct measurements of portal vein pressure, due to inconsistent availability of data in our routine clinical care sample. Importantly, our study design does not allow us to establish causality. The potential for unmeasured confounders or confounding by indication—particularly with relation to treatment of varices—cannot be conclusively eliminated.

Our analysis also has a number of unique strengths, including two large, well‐characterized samples that are among the largest to consider risk factors for PVT within specific liver disease etiologies and cirrhosis severity; our PBC cohort may be the largest analysed to date. Moreover, all patients in our sample were receiving standard clinical care for cirrhosis, which recommends regular imaging to screen for hepatocellular carcinoma roughly every six months, permitting identification of PVT even in the absence of symptoms or complications. Despite small variations in how often individual patients may receive these screening tests, we do not believe that any systematic bias is likely to have influenced our results. We have also applied a novel time‐dependent analysis that accounts for the evolution of patients' clinical and treatment status over time. For example, patients who began observation with compensated cirrhosis but progressed to decompensated cirrhosis or those who were untreated but then achieved SVR would still be included in this analysis.

In conclusion, neither SVR after antiviral treatment for HCV nor response to UDCA treatment for PBC was associated with risk of PVT in our sample. Notably, we found that treatment with NSBBs was associated with higher rates of PVT among patients with cirrhosis subsequent to either HCV or PBC. Because our study design does not allow us to draw conclusions about causality, additional prospective studies with more detailed characterization of cirrhosis stage—particularly factors such as hepatic venous pressure and velocity—as well as longer follow‐up are needed to confirm our observations.

## Author Contributions

Conception or design of the study: Gonzalez HC, Gordon SC, Lu M; acquisition, analysis, or interpretation of data: Gonzalez HC, Gordon SC, Trudeau S, Wu T, Rupp L, Melkonian C, Schmidt MA, Daida YG, Sahota AK, Bowlus CL, Lu M; drafting/reviewing of manuscript: Gonzalez HC, Gordon SC, Trudeau S, Wu T, Rupp L, Melkonian C, Schmidt MA, Daida YG, Sahota AK, Bowlus CL, Lu M; final approval of the version to be published: Gonzalez HC, Gordon SC, Trudeau S, Wu T, Rupp L, Melkonian C, Schmidt MA, Daida YG, Sahota AK, Bowlus CL, Lu M.

## Ethics Statement

The data for this analysis were drawn from the Chronic Hepatitis Cohort Study and the Fibrotic Liver Disease Study; both follow the guidelines of the US Department of Health and Human Services for the protection of human subjects. Study protocols were approved by the Institutional Review Board of each participating site.

## Consent

Requirements for written informed consent were waived due to the observational and de‐identified nature of the data.

## Conflicts of Interest

The authors declare no conflicts of interest.

## Supporting information


**Table 1** List of non‐selective beta blockers (NSBBs), statins, anti‐coagulants, and anti‐platelets (categorized with anti‐coagulants) included in analysis.

## Data Availability

The data that support the findings of this study are available from the corresponding author upon reasonable request.
