# Supplementary material for: Impact of Disease‐Specific Treatment and Non‐Selective Beta Blockers on Risk of PVT in Cirrhotic Patients With HCV or PBC
Source: Liver Int. 2026 Apr 3;46(5):e70628. doi: 10.1111/liv.70628 (PMC13049362; doi:10.1111/liv.70628)
Supplement: Supplementary file 1 — Table 1 List of non‐selective beta blockers (NSBBs), statins, anti‐coagulants, and anti‐platelets (categorized with anti‐coagulants) included in analysis. [file LIV-46-0-s001.docx]

**Supplementary table 1: List of non-selective beta blockers (NSBBs), statins, anti-coagulants, and anti-platelets (categorized with anti-coagulants) included in analysis.**

| **NSBBs** | **Statins** | **Anticoagulants** | **Antiplatelets** |
| --- | --- | --- | --- |
| carvedilol | atorvastatin | apixaban | aspirin |
| labetalol | ezetimibe/ simvastatin | dabigatran | clopidogrel |
| nadolol | lovastatin | enoxaparin | dipyridamole |
| propranolol | pravastatin | fondaparinux |  |
| sotalol | rosuvastatin | heparin |  |
|  | simvastatin | rivaroxaban |  |
|  |  | warfarin |  |
